# *Listeria monocytogenes* Biofilms in Food-Associated Environments: A Persistent Enigma

**DOI:** 10.3390/foods12183339

**Published:** 2023-09-06

**Authors:** Lawrence Finn, Helen Onyeaka, Sally O’Neill

**Affiliations:** School of Chemical Engineering, University of Birmingham, Birmingham B15 2TT, UK

**Keywords:** *Listeria monocytogenes*, persistence, biofilm, food-associated environment, whole-genome sequencing, genetic markers

## Abstract

*Listeria monocytogenes* (LM) is a bacterial pathogen responsible for listeriosis, a foodborne illness associated with high rates of mortality (20–30%) and hospitalisation. It is particularly dangerous among vulnerable groups, such as newborns, pregnant women and the elderly. The persistence of this organism in food-associated environments for months to years has been linked to several devastating listeriosis outbreaks. It may also result in significant costs to food businesses and economies. Currently, the mechanisms that facilitate LM persistence are poorly understood. Unravelling the enigma of what drives listerial persistence will be critical for developing more targeted control and prevention strategies. One prevailing hypothesis is that persistent strains exhibit stronger biofilm production on abiotic surfaces in food-associated environments. This review aims to (i) provide a comprehensive overview of the research on the relationship between listerial persistence and biofilm formation from phenotypic and whole-genome sequencing (WGS) studies; (ii) to highlight the ongoing challenges in determining the role biofilm development plays in persistence, if any; and (iii) to propose future research directions for overcoming these challenges.

## 1. Introduction

*Listeria monocytogenes* (LM) is a Gram-positive bacillus responsible for the foodborne illness listeriosis [1,2,3]. Most listeriosis cases are sporadic, but significant outbreaks have also been reported [4,5,6]. These have been associated with a whole range of foods—most often ready-to-eat foods, such as sliced cooked meats, pork pies, crab meat and pate [7,8,9]. While the incidence of listeriosis is relatively low, it is a significant concern to public health due to its high mortality (20–30%) and hospitalisation rates [3,10]. The disease is especially dangerous in the elderly, immunocompromised, newborns, pregnant women and their unborn infants—causing septicaemia, meningitis, miscarriage and stillbirth [1,7].

LM is ubiquitous in the environment and able to grow under a range of adverse conditions, including a wide temperature (−0.4 to +45 °C) and pH range (4.4–9.4) and low a_w_ (down to 0.90) [3]. This makes it well adapted to growth and survival in food-associated environments [11]. There are 14 LM serotypes, with serotype 4 h being the most recently recognised [4]. However, >95% of human infections are caused by just three serotypes (1/2a, 1/2b, and 4b) [12]. The serotypes are divided into four phylogenetic lineages (I-IV) [13]. These, in turn, are subdivided into sequence types (STs) and clonal complexes (CCs) by multilocus sequence-typing (MLST)—with most isolates belonging to lineages I and II [14].

It is well established that LM can persist in a range of food-associated environments for months or years, despite cleaning and disinfection [15,16,17,18]. While there is no standardised definition of persistence, it is generally used to describe the long-term survival of a bacterial strain in a food-associated environment over time; it is based on recurrent isolation of listerial strains from a facility on different dates over a defined period, which are found to belong to the same molecular subtype [4,18]. Persistent strains have been recovered from a range of food and non-food contact surfaces, such as tables, floors and drains, as well as equipment, utensils and raw materials (among others) [18]. The exact criteria used to establish persistence, such as the number of isolation events, time frame or choice of subtyping method, vary widely between studies and are often subjective [17,18,19]. Therefore, to avoid excluding potentially relevant studies, this review will adopt the definitions employed by the authors of the included studies. Additionally, the term ‘presumed non-persistent’ (PNP) is used to denote any strain not observed to persist in a given facility, as proposed by Kastbjerg and Gram [20], in order to account for the possibility that they might be persistent in other settings.

The persistence of LM poses a serious food safety concern due to its potential to result in food contamination [8,18]. This has been implicated in several devastating listeriosis outbreaks [8,15]. One of the most renowned occurred in the US in 2000 [21]. It involved 29 cases and four deaths and was caused by an LM strain that persisted in a meat processing plant over 12 years. Given the high levels of contamination and exposure usually required for a given subtype to cause infection, it has been suggested that most listeriosis outbreaks may be caused by persistent LM subtypes repeatedly contaminating products over prolonged periods [18]. Persistent product contamination is also burdensome to food businesses and economies, resulting in costly recalls, withdrawals, seizures, closures and reputational losses [18]. In the US alone, the estimated annual cost of recalls caused by LM is USD 1.2–2.4 billion [22], while the estimated annual cost of illness associated with listeriosis is USD 2.6 billion [23]. Therefore, it is essential to develop more targeted methods to control and prevent the persistence of this pathogen in order to improve food safety and reduce the associated economic and food business costs.

The mechanisms responsible for listerial persistence are not well understood [18,24]. Some have hypothesised that persistence might be due to increased tolerance towards commercially used disinfectants or enhanced adaptation towards some of the stresses encountered in food-associated environments [25,26]. However, a lack of clear evidence has been found to support this; this has led some to postulate that persistence is mainly due to a failure of cleaning and sanitation procedures, as opposed to any unique strain properties [17,18]. One of the most widely explored hypotheses is that persistent strains are more capable of producing biofilms on abiotic surfaces in food-associated environments [27,28,29]. While previous reviews have failed to find clear evidence to support this hypothesis due to conflicting research findings, they were not comprehensive and were missing data from several phenotypic studies [4,16,17,18,24]. Nor did they discuss many of the more recent studies that have investigated persistent strains for evidence of enhanced biofilm production using whole-genome sequencing (WGS) techniques.

This review explores the current state of knowledge on the role of biofilms in the persistence of LM. It comprehensively summarises the available research from phenotypic and WGS studies on the relationship between listerial persistence and biofilm development. It also highlights some of the major challenges that remain in understanding the role biofilm development plays in persistence, if any, and discusses potential strategies for overcoming them. Unravelling the enigma of what allows this pathogen to persist will be critical for developing effective control and prevention measures that can help prevent it from being transmitted to humans. By discussing challenges and ways of overcoming them, this review aims to provide valuable insights for researchers, policymakers and food industry professionals working to improve food safety and prevent foodborne illness.

## 2. LM Biofilms

**Figure 1:**
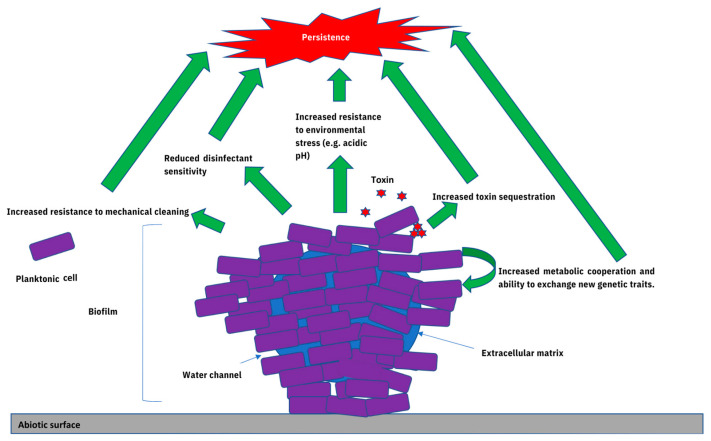
Schematic diagram of a mature *Listeria monocytogenes* (LM) biofilm with a three-dimensional mushroom shape. Mature biofilms formed under field conditions may contain other microorganisms [1]. Green arrows represent some of the main survival advantages associated with cells within the biofilm compared with their planktonic counterparts, which may allow them to persist in food-associated environments [40,41,42].

A biofilm is a ‘community of microbes associated with a surface, typically encased in an extracellular matrix’ (EM) (Figure 1) [30]. This is consistent with the mix of biofilm morphologies described for LM. Dependent on the strain and environmental conditions, these range from knitted chains and dense, three-dimensional structures resembling mushrooms or honeycombs, to cellular monolayers—with little or no EM [29,31,32,33]. The range of surfaces on which listerial biofilms may develop is equally varied and includes stainless steel, glass, rubber and plastics (among others) [29,31,34,35]. It is unclear if the lack of EM reported in LM biofilms results from microscopy sample preparation or the experimental conditions [13]. However, the EM of LM biofilms mainly comprises extracellular DNA, proteins and exopolysaccharides—particularly teichoic acids [36].

**Figure 2:**
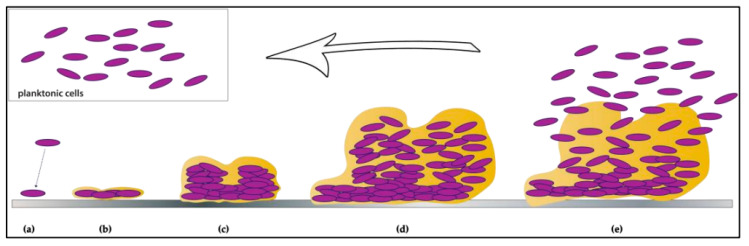
Diagram representing the key stages of biofilm development (image adapted from Colagiorgi et al. [1] under the terms and conditions of the Creative Commons Attribution (CC BY) license, http://creativecommons.org/licenses/by/4.0/ (Accessed 21 June 2023)). (**a**). Planktonic cells begin by adhering reversibly to a surface via Van der Waals forces and other weak interactions between cell surface structures and the material they are in contact with [2]. (**b**). The cells begin to establish stronger interactions between themselves and the surface, such as covalent bonding. These interactions are mediated by cellular appendages and by deposition of extracellular matrix (EM). Adherence becomes irreversible. (**c**). The adherent cells multiply and recruit other cells from the surrounding environment, producing microcolonies. (**d**). Eventually, a mature biofilm is produced, characterised by a complex three-dimensional structure with its own ecosystem. It includes pores for nutrient exchange and waste removal (**e**). Cells within the biofilm disperse, returning to a planktonic state, which allows them to colonise new surfaces.

Biofilm development is a multi-staged process, beginning with adherence to a surface (Figure 2). Adherence is reversible within minutes to hours of surface contact, relying on a combination of physical forces and/or bacterial appendages, such as flagella [37]. It is mediated by weak interactions, such as Van der Waals interactions, electrostatic forces and hydrophobic interactions [16]. During this phase, bacterial cells still exhibit Brownian motion and can easily be removed—for example, by rinsing [38]. However, stronger interactions eventually develop between the cells and the surface, such as covalent and ionic bonding, mediated by exopolysaccharide fibrils, flagella and/or other surface appendages. At this point, adherence is considered irreversible, as the cells can only be removed by shearing forces, such as scrubbing or scraping, or by chemical breaking of the attachment forces, such as by enzymatic, detergent and/or heat treatment [37]. These cells then multiply and produce EM, microcolonies and eventually, multi-layer and multi-species communities [39,40]. The biofilm reaches maturity when a three-dimensional structure is achieved, complete with water channels supporting nutrient and metabolite exchange and waste elimination [2]. Depending on the microorganism and experimental conditions, this can take up to 10 days or more to occur in vitro [2]. Cells within the biofilm, possibly in response to nutrient limitation, may eventually disperse and go on to colonise new niches [2].

A range of environmental factors can influence listerial biofilm development. These have been discussed extensively elsewhere, and so are touched upon only briefly here [1,13,16,41]. One of the main factors appears to be temperature [1]. Studies generally report that LM adherence and biofilm formation increases as temperature increases up to 30–37 °C [42,43,44,45,46,47], possibly due to changes in cell surface properties, such as hydrophilicity [35,48]. The properties of the surface material also have an important effect on listerial biofilm development, such as topography [16]. Rougher surfaces, particularly worn or corroded ones, provide more opportunity for biofilm formation, as surface defects can trap nutrients and water—facilitating bacterial proliferation [37,49]. They may also provide protection from cleaning and disinfection [50]. This makes stainless steel an ideal material for fabricating food processing equipment and utensils, as its hardness, corrosion resistance and ability to be polished help to ensure a smooth surface [51]. Other metals and metal nanoparticles, such as copper and silver, also have intrinsic antibacterial properties and can inhibit LM biofilm formation [52,53]. Therefore, they are also of interest for use within the food industry. Several studies have observed faster and higher rates of biofilm formation on hydrophilic surfaces, such as stainless steel, compared with hydrophobic materials, such as polystyrene [35,41,45,54,55]. However, opposite results have also been reported [56,57,58]. These conflicting findings may reflect differences in the test strains and/or experimental designs [16].

Other important factors that can influence listerial biofilm development include pH, NaCl concentration and nutrient availability [13,16]. The attachment of LM to stainless steel was found to be significantly greater in suspensions containing 0.15 M, compared with 0.0015 M NaCl [43]. It has been hypothesised that this may have resulted from alterations in cell surface hydrophobicity [59,60]. However, in another study, the adherence of LM was not significantly different when cultured in general-purpose media containing 0% or 6% NaCl (*w*/*w*) and even showed a significant decrease in media containing 11% NaCl, suggesting that high NaCl concentrations may suppress listerial biofilm formation [61]. Several studies have also shown that more acidic conditions, created by adding citric or lactic acid to the growth medium, can promote the attachment of LM Scott A to stainless steel; this may be due to the protonation of negative groups on the bacterial cell surface [43,62]. However, contrasting results were reported by Smoot and Pierson [48,54], who found that LM Scott A colonised stainless steel best at pH 7.0. There is a similar lack of consensus within the literature as to the optimal nutrient conditions for LM biofilm development. While some studies have found that nutrient limitation decreases LM biofilm formation [35,56,63,64], others have found the opposite effect [65,66]. It has been suggested that the ability of LM to form biofilms under different nutrient conditions may be strain and surface-material-dependent [67,68,69], which could explain some of these contradictory findings [13].

Lastly, interactions with other microorganisms can also have an important effect on listerial biofilm formation. For example, *Pseudomonas* spp. are commonly encountered on surfaces in food-associated environments and have been shown to promote or inhibit LM biofilm formation, dependent upon the strain or conditions [16,70,71].

## 3. The Contribution of Biofilms towards Microbial Persistence within Food-Associated Environments

There are several ways in which enhanced biofilm production might contribute towards the persistence of microorganisms within food-associated environments. Firstly, biofilm production represents an important survival strategy for many microorganisms. Compared with their planktonic counterparts, biofilm-associated bacteria can be difficult to remove mechanically from surfaces and are known to exhibit marked reductions in sensitivity towards chemical disinfectants, which may allow them to resist conventional cleaning methods [51]. Secondly, biofilm formation may help bacterial cells adapt to other environmental stresses encountered in food-associated environments, such as high temperature, acidic pH, desiccation, UV and high salinity [72]. Thirdly, biofilm cells may be more capable of sequestering toxins, metabolic cooperation and nutrient exchange [73]. They may also be more capable of acquiring new genetic traits, such as antimicrobial resistance genes, which might contribute towards survival in food-associated environments. Indeed, biofilm formation appears to play a role in the persistence of several important bacterial foodborne pathogens within food-associated environments, including *Salmonella enterica* [74,75], *Escherichia coli* [76] and *Campylobacter* spp. [77]. Each will be considered in turn.

Non-typhoidal *S. enterica* is a Gram-negative bacillus and a common cause of foodborne gastroenteritis, usually associated with poultry products [78]. However, contaminated meat, fresh produce, nuts, spices, flour, milk and drinking water can also act as vehicles [78]. Like LM, *S. enterica* is able to form biofilms on a range of surface materials and to persist in food-associated environments, such as poultry slaughterhouses [79]. Vestby et al. [74] reported that the biofilm-forming ability of 111 *Salmonella* strains isolated from feed and fish meal factories was correlated with their persistence within the factory environment. Similar findings were reported by Dantas et al. [75], who identified a relationship between biofilm formation and the persistence of 40 *Salmonella* strains isolated from a poultry slaughterhouse. Unlike LM biofilms, *Salmonella* biofilms may express an abundance of EM, consisting largely of cellulose and curli fimbriae, which can help them to survive environmental stressors, such as desiccation and disinfectants [80,81]. This is believed to be the major reason for their ability to persist in food-associated environments [79]. Other factors involved in *S. enterica* biofilm formation include flagella, surface-associated proteins, such as Bap, and quorum-sensing (QS) systems, such as LuxS [78,82].

*E-coli* is a Gram-negative bacillus, which is closely related to *S. enterica* [78]. While most strains are non-pathogenic, some are highly pathogenic. For example, the Shiga-toxin-producing strain *E-coli* O157:H7 can cause haemorrhagic colitis and potentially life-threatening haemolytic uraemic syndrome [78]. *E-coli* outbreaks are associated with the consumption of raw meat or meat products, as well as fresh produce [78]. The organism was found to persist on a conveyor belt in a meat packing plant, surviving daily cleaning and disinfection practices [83]. Beef-production companies have also been known to experience periods of increased product contamination caused by *E-coli* O157:H7 [76], most likely due to persistent contamination of the processing environment [84]. Compared with control strains, the *E-coli* strains isolated during these periods have been found to exhibit high levels of biofilm formation on food contact surfaces, linked to higher copy numbers of the pO157 plasmid and reduced susceptibility to common sanitisers [76]. Like *S. enterica*, curli fimbriae and cellulose are also major components of *E-coli* biofilms, likely to play a similar role in facilitating persistence [78]. However, fimbriae and flagella can also enhance *E-coli* biofilm formation [85] and may contribute towards persistence.

*Campylobacter* spp. are Gram-negative, microaerophilic bacilli [82]. They are the common cause of bacterial gastroenteritis globally, particularly *C. jejuni* and *C. coli* [86]. Like *Salmonella*, infection is usually associated with poultry meat, as well as other foods, such as raw milk [82]. The organism requires specific conditions to grow, such as a microaerophilic atmosphere and a temperature between 37 °C and 42 °C [82]. It is also sensitive to food-processing conditions, such as acidic pH [82]. Despite this, there is evidence that it can persist within food-associated environments, such as farms and dairies, by forming biofilms [86]. For example, a *C. jejuni* outbreak in Finland was linked to a persistent strain contaminating bulk tank milk over at least seven months [77]. The outbreak strain was able to form biofilms under laboratory conditions, unlike a closely related strain from a separate dairy. This led the authors to conclude that biofilm formation on the milking machine or milk tank may have been responsible for the persistence of the outbreak strain. Similar to LM [4], the mechanisms involved in *Campylobacter* biofilm formation have not been fully elucidated [82]. However, various factors appear to be involved, such as flagella, surface proteins and QS systems [77].

Given the benefits associated with biofilm formation and the role that it seems to play in the persistence of other foodborne pathogens, it is plausible that this might also contribute towards the persistence of LM, as has been hypothesised.

## 4. Phenotypic Comparisons of Biofilm Development in Persistent and PNP LM Strains

Numerous studies have compared the biofilm development of persistent and PNP LM strains (summarised in Supplementary Table S1). However, compared with other persistent foodborne pathogens, such as *S. enterica*, the evidence of whether there is a relationship between biofilm formation and persistence in this species has been mixed. 

Several studies have reported evidence of enhanced biofilm formation among persistent LM strains [19,27,29,40,87,88,89,90,91]. For example, Norwood and Gilmour [27] measured the adherence to stainless steel coupons of 32 persistent and 25 PNP strains isolated from farms and food processing plants. After 24 h of surface contact at 25 °C, mean plate counts on the coupons were significantly higher for the persistent strains. Similar results were obtained by Lunden et al. [87] after they compared the adherence of a persistent strain to that of three PNP strains isolated from meat dicing plants on stainless steel coupons over two hours at 25 °C using epifluorescence microscopy counts. Borucki et al. [29] tested the biofilm development of 11 persistent and 15 PNP strains isolated from bulk milk tanks at a dairy to polyvinyl chloride (PVC) microtiter plates using crystal-violet staining. After 40 h of surface contact at 30 °C, the mean biofilm level of the persistent strains was significantly greater than that of the PNP strains. Scanning electron microscopy (SEM) was also used to examine the attachment of one strain from each group to stainless steel and PVC coupons incubated under the same conditions. While the PNP strain adhered mostly as sparse cellular aggregates on stainless steel and single cells on PVC, the persistent strain adhered to both materials as a dense, three-dimensional composite with water channels, consistent with a biofilm.

A similar number of studies have reported no apparent difference between the biofilm formation of persistent and PNP LM strains [47,56,92,93,94,95,96,97,98,99,100,101]. For example, Djordjevic et al. [56] compared the biofilm formation of five persistent and five PNP strains isolated from fish processing plants to PVC microtiter plates using the crystal-violet method; after 40 h of surface contact at 32 °C, no relationship was observed between the biofilm levels of the persistent and PNP strains. Similarly, Harvey et al. [92] compared the biofilm formation of 36 persistent strains recurrently isolated from various food processing environments to that of 32 PNP strains. However, both exhibited comparable levels of biofilm formation, based on a 48 h polystyrene microtiter plate assay and a 14-day Petri-dish assay with crystal-violet staining. Jensen et al. [93] used a similar 48 h microtiter plate method to compare the adherence of 10 persistent and eight PNP strains isolated from fish processing plants on polystyrene. Despite supplementing the growth medium with up to 5% NaCl (*w*/*v*) to simulate the fish processing environment, no systematic differences were observed between persistent and PNP strains after 48 h of surface contact at 37 °C. Lee et al. [44] also failed to identify any significant differences in biofilm formation between persistent and PNP strains in media supplemented with NaCl (0.85% (*w*/*v*)).

Factors that may explain the inconsistent findings between studies could include differences in experimental conditions, such as incubation temperature, surface material and nutrient availability [1,13,16,41]. Similar factors are known to influence biofilm development in other foodborne pathogens, such as *S. enterica* or *Campylobacter* spp. [79,82]. Due to the different combinations of strains and culturing conditions used, it is difficult to compare the results of these studies in order to identify any specific environmental conditions that may be associated with enhanced biofilm formation in persistent LM strains, such as the type of surface material. However, based on individual studies where the effect of altering a single experimental condition was investigated, there is evidence that several different environmental factors—or a complex interplay between them, may influence the biofilm-forming ability of persistent strains, such as surface contact time.

Lunden et al. [28] compared the adherence to stainless steel of three persistent strains with 14 PNP LM strains isolated from poultry and ice cream plants over different surface contact times. After 1–2 h of surface contact at 25 °C, the persistent strains exhibited significantly greater adherence, based on epifluorescence microscopy counts. However, after 72 h, 50% of the PNP strains exhibited similar or equal levels of adherence. Other studies have reported that persistent strains were significantly better at surface colonisation than PNP strains using the crystal-violet method but only as surface contact time was increased from 24 to 48 h, based on plate counts, [89]—or only as the temperature was increased within the 21–37 °C range [91,102]. These findings suggest that temperature, as well as surface contact time, may influence the biofilm formation of persistent LM strains. However, others have found no significant differences in biofilm formation between persistent and PNP strains on polystyrene or stainless steel over a range of incubation temperatures (10–40 °C) [47,97]. This may have been due to differences in strains and/or other experimental conditions.

The biofilm formation of persistent LM strains has also been found to vary according to the composition and concentration of the growth medium [19,44,103] and the surface material [104]. For example, Lee et al. [44] found that persistent strains exhibited significantly increased biofilm formation compared with PNP strains, but only in neat (undiluted), as opposed to dilute (1:10), brain heart infusion (BHI) broth. Magalhaes et al. [104] also observed significantly higher biofilm formation among persistent, compared with PNP strains using the crystal–violet method but only on stainless steel or silicon rubber, as opposed to PVC or polystyrene. Opposite results were obtained by Costa et al. [96], who reported that persistent strains exhibited significantly lower biofilm formation on stainless steel compared with polystyrene.

In addition to different experimental conditions, there are several other factors that might account for the conflicting results between studies investigating the relationship between biofilm formation and persistence. These include differences in the choice of biofilm assay, sample size, persistence definitions, and/or test strain properties, such as lineage or serotype [4,29]. Differences in biofilm formation according to serotype have been reported in *E-coli*, with serotype O103:H2 isolates shown to produce more biofilm at 12 °C, 20 °C and 37 °C, compared with strains representative of serotypes O26:H11 or O103:H25 [105]. Several studies have also suggested a relationship between biofilm production and lineage or serotype in LM. For example, Norwood and Gilmour [27] reported that serotype 4b strains formed higher levels of biofilm on stainless steel compared with serotype 1/2a strains. However, opposite results were achieved by Nilsson et al. [47] and Upham et al. [106], who found that serotype 1/2a strains exhibited the highest levels of biofilm formation on stainless steel compared with serotype 4b. Similar results were reported recently by Park et al. [107]. Contrastingly, Keeney et al. [108] observed the highest levels of biofilm production among serotype 1/2b strains, which they suggested was due to possession of SSI-1, as this was present among most of the serotype 1/2b strains but few of the weakest biofilm-producing strains, belonging to serotype 4b.

Investigations into whether lineage may be a reliable predictor of biofilm formation in LM have also produced conflicting results. Takahashi et al. [109] and Djordjevic et al. [56] observed higher levels of biofilm formation among lineage I, compared with lineage II LM isolates on PVC. However, opposite results were reported during some studies [29,64,92,110,111]. Genotype (ST or CC) may be a more reliable predictor of listerial biofilm production than lineage or serotype, as Lee et al. [44] observed. However, while some studies have identified CC-specific differences in biofilm production under some conditions [107,112], further research is required to clarify the relationship between genotype and biofilm formation in persistent LM strains.

Overall, the results of phenotypic studies investigating the relationship between biofilm production and persistence are highly conflicting and cannot be clearly explained by differences in lineage or serotype. Therefore, it remains unclear whether persistent strains exhibit enhanced biofilm formation.

## 5. Genetic Mechanisms Involved in Biofilm Development in LM

With advances in WGS techniques, a few studies have compared the genomes of persistent and PNP LM strains to identify genetic markers that may help to explain, identify or predict persistence. This includes markers associated with biofilm production (summarised in Table S1). The following section provides an overview of the functions of some of the main genetic loci associated with biofilm formation in LM, which have been investigated for an association with persistence in WGS studies, before going on to discuss the key findings of these studies.

One of the main biofilm-associated genes in LM is *flaA*, which encodes the main structural component of the bacterial flagella, flagellin A [113]. Flagella-mediated motility appears to promote initial attachment under both static and dynamic conditions but is not essential for—and may even inhibit—full biofilm formation under dynamic conditions [114,115,116,117]. Expression of *flaA* in LM is controlled by at least five other regulatory genes [113]. Some of these genes have also been shown to be required for effective biofilm development, which may be due, in part, to their effect on flagella expression and motility. These include the positive regulatory factor A gene, *prfA,* involved in the later stages of biofilm development [118,119], and the degradation enzyme regulator gene (*degU*), involved in adherence to plastic surfaces [120,121]. Both are also involved in virulence.

The actin-assembly-inducing peptide precursor (ActA), encoded by *actA*, is another important virulence determinant in LM, which promotes bacterial aggregation and biofilm development [122]. Compared with wild-type (WT) strains, Δ*actA* mutant strains exhibit significant reductions in biofilm development on glass under both static and dynamic conditions due to reduced clustering. The transcriptional regulator of stress response genes, SigB, is also required to reach WT biofilm levels under both static and continuous-flow conditions on stainless steel and polystyrene [123,124]. However, it may only be involved in the later stages of biofilm development, as no difference in biofilm levels was observed between WT and SigB-deficient strains after 24–40 h of surface contact [116,123]. Contrastingly, the biofilm-associated protein (BapL), encoded by *bapL*, appears to be involved in adherence in some LM strains, but its exact role in biofilm development is not well understood [113,125]. Strains naturally lacking *bapL* may exhibit either enhanced or reduced adherence to polystyrene and stainless steel, independent of serotype, and both may still go on to produce biofilms [126].

The listerial virulence proteins internalins A and B (InlA and InlB) have also been implicated in listerial biofilm development [125]. Deletion of *inlA* or *inlB* in LM EGD-e has been associated with significant reductions in adherence to glass compared with a WT strain, especially when both genes are deleted [127,128]. However, *inlA* truncation has been associated with increased biofilm production compared with WT strains [129]. The truncated protein lacks the cell wall-binding domain and is secreted where it undergoes proteolytic cleavage and may contribute towards the biofilm EM. Internalin L (InlL) appears to play a similar role in listerial adherence, as deletion of *inlL* has been associated with reduced attachment to polystyrene [130].

The *agrBDCA* operon is a peptide-based QS system in LM that plays an important role in biofilm development [131,132,133]. It consists of four genes, including *agrC*, *agrA*, *agrD* and *agrB*, respectively encoding a histidine kinase (AgrC), a response regulator (AgrA), a precursor peptide (AgrD) and a protein (AgrB), which converts the precursor peptide into an autoinducing peptide. LM Δ*agrA* and Δ*agrD* deletion mutants both exhibit significant reductions in adherence to glass or polystyrene surfaces compared with WT strains within the first 24 h of surface contact [131,132]. However, whether LM relies on *agrBDCA* to coordinate biofilm development via QS has been called into question, due to limited evidence that the operon responds to changes in listerial cell density [134].

The LuxS system is another QS system in LM that has been implicated in biofilm development [11,113]. It encodes a protein (LuxS) involved in producing autoinducer-2 (AI-2) precursor molecules [55]. LM strains with mutations in *luxS* have been known to produce denser biofilms and to attach more readily to glass compared with WT strains [135,136]. Other notable genes implicated in listerial biofilm development include *recO* and *lmo2504*. The former encodes a DNA repair and protection protein (RecO), while the latter encodes a putative cell-wall binding protein (Lmo2504), which may also be involved in chromosomal maintenance; both have been shown to be overexpressed in biofilm-associated, compared with planktonic cells [137,138].

An association has also been reported between LM strains possessing a prophage insertion in the *comK* gene and enhanced biofilm production [139]. Significantly, one of the strains in this study that produced the highest cell density was a putatively persistent strain isolated from a US meat processing plant [21]. This strain also carried a *comK* prophage insertion, suggesting that this may have helped it to persist. The Genetic determinants involved in biofilm formation in LM are shown in Table 1.

## 6. WGS Comparisons of Biofilm Production in Persistent and PNP LM Strains

One of the most comprehensive attempts to identify persistence markers was conducted by Nielsen et al. [140]. Based on a literature search, the authors identified 15 genes which might play a role in persistence. Six of them were chosen due to evidence of involvement in biofilm production, including *actA*, *bapL*, *recO*, *lmo2504*, *luxS* and the flagellar operon gene, *lmo0673*. They used WGS to compare the genomes of 21 persistent and 17 PNP strains isolated from pork and salmon-processing plants to see whether any of these genes were overrepresented among persistent strains. However, none of the genes examined were clearly linked to persistence, with equal representation among persistent and PNP isolates. The genes were also examined for any insertions, deletions or single nucleotide polymorphisms (SNPs) that might distinguish persistent LM strains, but none were identified. Similar results were reported by Muhterem-Uyar et al. [141], who examined the genomes of six persistent strains and five PNP strains isolated from an Austrian cheese processing plant for the presence of genes associated with survival in food-associated environments. Twenty-eight of them were genes associated with biofilm development, including *lmo2504*, *flaA*, *agrD*, *agrA*, *degU*, *luxS*, *bapL* and *agrA* (among others). However, these genes were largely present in both persistent and PNP strains.

Palaiodimou et al. [142] compared the genomes of four persistent and four PNP LM strains isolated from an Irish meat processing plant with respect to several markers previously shown to have been linked to biofilm production. These included the virulence markers *inlA*, *inlB*, *inlL*, *prfA* and *actA*. The persistent strains were more likely to harbour a truncated *inlA* gene, previously associated with increased biofilm production [129,142]. One of the persistent strains was also unique in possessing a premature stop codon (PMSC) in *prfA*, although this mutation was not shared by the other persistent strains and its effect on biofilm phenotype was not investigated [142]. However, none of the markers could universally explain these strains’ persistence [142].

Fagerlund et al. [143] sequenced and compared the genomes of 551 persistent and 218 PNP LM isolates from Norwegian food, food-associated environments and raw materials using a combination of whole-genome SNP and whole-genome MLST analyses. No association was found between persistence and *inlA* truncation. However, the authors identified a weak but statistically significant association between persistence and possession of the biofilm-associated genes *bapL* and *inlL*. Opposite results were reported by Yi et al. [144], who compared the genomes of 96 persistent LM CC554 isolates with those of 60 PNP isolates from three commercial apple packinghouses for the presence of specific genes associated with survival in food-associated environments. This included *inL* and *bapL* but also *lmo0673*, *lmo2504*, *luxS* and *recO*. However, the persistent isolates did not contain any biofilm-associated genes that were not present in the PNP isolates, and none of the persistent isolates possessed *bapL* or *inlL*. Other WGS studies have also failed to identify any clear relationship between persistence and possession of any genetic markers linked to biofilm production [89,145,146,147].

Nielsen et al. [140] have suggested that differences in the accessory genome may be associated with persistence. Indeed, Fagerlund et al. [148] hypothesised that plasmids and prophages may have contributed towards the persistence of six ST8 strains in fish and poultry processing plants after identifying a high level of conservation between these elements within each strain. A similar hypothesis was proposed by Schmitz-Esser et al. [149] and Muhterem-Uyar et al. [141] after they observed a high level of plasmid and prophage conservation among persistent ST121 and ST5 strains, respectively. In addition, several studies have investigated whether a *comK* prophage insertion may be a potential persistence marker, due to a possible association with enhanced biofilm production [139,141,142,149,150]. However, none have found a clear relationship between persistence and possession of a *comK* prophage. Nowak et al. [89] also failed to identify any relationship between persistence and plasmid or prophage content in LM.

Overall, WGS studies have failed to identify any consistent genetic differences between persistent and PNP LM strains linked to biofilm production. This would appear to support earlier suggestions that persistence may be due to a failure of cleaning and sanitation procedures to remove LM from harbourage sites in food-associated environments, as opposed to any unique strain properties [17,18]. While environmental factors such as this may significantly contribute to persistence, this is not to say that some strains do not still harbour traits that increase their chances of persisting within food-associated environments [18,99]. Enhanced biofilm production may be just one of these, the importance of which may depend on the strain or environmental conditions or a complex interplay between the two. This may explain why some studies focusing on one trait, such as enhanced biofilm formation or disinfectant tolerance, have observed an association with persistence but not others [99].

Evidence that LM may use different mechanisms to persist in different niches was provided by Cherifi et al. [99]. The authors observed that persistent isolates from a slaughterhouse conveyor belt were more likely to exhibit reduced sensitivity towards quaternary ammonium compound (QAC) disinfectants than persistent strains isolated from difficult-to-clean equipment, due to possession of the *bcrABC* cassette. The authors hypothesised that the conveyor belt strain, which was likely to have had the greatest disinfectant exposure, had persisted due to QAC tolerance, while the equipment strain lacking the same cassette had persisted due to other mechanisms, such as biofilm formation. A similar situation has been observed in *Salmonella*. Compared with poultry isolates, *Salmonella* isolates from produce exhibited a greater ability to form biofilms on food-contact materials, suggesting that this may have allowed these strains to adapt to and persist on surfaces within the produce supply chain [151].

## 7. Challenges and Solutions

Some important challenges remain in understanding whether enhanced biofilm formation plays a role in listerial persistence. These may well have contributed towards some of the conflicting results identified in this review. If a clearer understanding of persistence mechanisms is to be achieved, these challenges will need to be addressed. Each will be considered, in turn, along with proposed solutions.

### 7.1. Lack of a Standardised Persistence Definition

One of the main challenges in studying persistence is the lack of a standardised definition [17,18]. This was particularly apparent among some of the studies identified in this review. Persistence definitions ranged from the isolation of ‘an identical PFGE pattern…three times from the same store and site over a six-month period’ [152] to the isolation of the same MLST genotype at least three times over 1–5 years from the same food premise [*sic*]’ [44]. Such definitions will also be heavily influenced by choice of sampling protocol and its ability to detect recurrent strains in food-associated environments [29]. The use of such different criteria to define persistence makes direct comparison of the results difficult and could have contributed towards the conflicting results. Therefore, a more standardised persistence definition is needed. During this review, one of the most common persistence definitions was the isolation of the same subtype on two or more occasions over six months [19,89,90,94,102,152]. Therefore, while somewhat arbitrary, this may serve as a more standardised definition in future studies.

### 7.2. Possibility of Repeated Reintroduction 

One of the other main challenges in studying persistence is distinguishing it from the repeated reintroduction of the same subtype [18]. It is important to consider the discriminatory power of the subtyping method in this [18]. The use of a low-discriminatory technique may fail to distinguish persistence from random reintroduction of genetically similar but epidemiologically unrelated strains. This was a potential problem among some of the older studies identified in this review, which relied on subtyping methods such as ribotyping, restriction fragment length polymorphism (RFLP) and multilocus enzyme electrophoresis (MEE) [19,27,56,92]. These techniques do not offer the same level of resolution as PFGE, or, better still, WGS techniques [153]. 

The unparalleled discriminatory power of WGS techniques and their ability to define genetic distances means that they lend themselves well to helping distinguish persistence from the repeated reintroduction of the same subtype [143,153]. Stasiewicz et al. [145] were recently able to develop such a technique based on the assumption that a population of persistent isolates from the same source must have descended from a common ancestor following a single introduction, and so are likely to have significantly fewer median SNP differences among themselves compared with identical PFGE subtypes from other sources. This was consistent with what they observed when they compared the genomes of putatively persistent and PNP isolates from U.S. retail deli sites belonging to the same PFGE type. However, several LM strains from separate food premises were also identified with identical or near-identical genome sequences, consistent with other reports [145,154,155,156].

The finding that identical LM isolates may be found in apparently unrelated food premises highlights a need for contextual information about the premises themselves in order to be able to help distinguish persistence from repeated reintroduction [18]. Recurrent isolation of the same subtype from premises designed and operated to prevent bacterial contamination, such as through the use of pathogen-free ingredients or the use of Good Manufacturing Practice (GMP), is more likely to represent persistence than a premises that does not use such procedures [18]. Equally, recurrent isolation after sanitation procedures but before the reintroduction of equipment or other potential fomites is also likely to point towards persistence over repeated reintroduction [145]. This was a potential problem identified among studies identified in this review, many of which provided limited information about the premises from which their persistent strains were isolated. For example, of the studies that had compared biofilm formation between persistent and PNP strains, only four reported using persistent strains that were not detectable in raw materials [28,56,100,111]. Similarly, only one study reported isolating persistent strains after applying sanitation procedures [99]. This makes it difficult to rule out the possibility that the strains in these studies were repeatedly reintroduced.

The difficulty in distinguishing persistence from repeated reintroduction has been widely recognised [6,15,19]. Therefore, the lack of consideration given to the possibility of repeated reintroduction in many studies may reflect a lack of clarity among researchers as to how to distinguish the two, rather than a lack of awareness of the issue. To counteract this, future studies should use a high-discriminatory subtyping method, such as PFGE or WGS. They should also provide details about the premises themselves and the isolation procedure. This should include information about whether the ingredients were sampled to check for the presence of the persistent subtype, whether the premises adhered to GMP and whether the persistent subtype was recovered after sanitation procedures.

### 7.3. Assay Limitations

Most studies identified in this review relied exclusively on the crystal-violet assay to compare the biofilm development of persistent and PNP strains [40,44,47,90,91,92,93,94,97,98,99,100,102,103,152,157]. While there is no ‘gold standard’ technique for studying biofilms, this technique specifically measures biomass and cannot distinguish between viable cells and EM, nor can it reveal the morphology of the adherent mass [46,158]. As Kalmokoff et al. [159] observed, the presence of adherent LM cells does not necessarily culminate in a mature biofilm. Therefore, without microscopic examination to check for EM or other indicators of a mature biofilm, such as EM, water channels or a three-dimensional structure, it is impossible to determine whether surface colonisation has advanced beyond adherence [2,18]. The crystal-violet method also suffers from a lack of reproducibility and potential inaccuracies resulting from the detachment of biofilm cells during the washing steps [158]. Therefore, while it may offer a rapid preliminary measure of biofilm development, it should be complemented by microscopic methods and techniques capable of quantifying the number of viable cells within the biofilm, such as plate counts [158].

### 7.4. Experimental Conditions Unrepresentative of Food-Associated Environments 

In addition to suffering from limitations associated with their choice of assay, many of the studies identified in this review did not necessarily use experimental conditions representative of food-associated environments. For one, most relied on incubation temperatures of 30–37 °C [29,40,56,90,94,95,98,99,100,101,102,103,111,152,157,160]. While such temperatures may be conducive towards listerial biofilm development, many food-associated environments operate at lower temperatures than this [16,17]. Indeed, LM is known for its ability to grow at refrigeration temperatures, unlike other foodborne pathogens [3]. Similarly, the studies were conducted in monoculture. However, in food-associated environments, LM would usually co-exist with other microorganisms capable of influencing its surface-colonising ability, such as *Pseudomonas* spp. [18]. The biofilm formation of other foodborne pathogens, such as *S. enterica*, *C. jejuni* and *E-coli* has been shown to be affected by similar interspecies interactions [161,162,163]. Indeed, *Campylobacter* spp. tend to exist more often in the environment as mixed, rather than mono-species biofilms [82].

In addition, exposure to sub-inhibitory concentrations of QAC disinfectants, as might occur in food-associated environments due to dilution, has also been shown to promote biofilm formation in lineage II LM clones, such as CC121 [112]. Only two studies identified during this review compared the biofilm formation of persistent and PNP LM strains under such conditions [99,103]. However, both failed to find any clear relationship between persistence and biofilm development. The presence of food residues may also positively or negatively affect listerial biofilm development [139], as they can with other foodborne pathogens, such as *S. enterica* [82]. However, none of the studies identified in this review evaluated the effect of food residues on the biofilm formation of their test strains. Future phenotypic studies should try to simulate conditions in food-associated environments more closely, including temperature, the presence of disinfectant residues and resident microflora.

### 7.5. Lack of Standardised Experimental Protocols

One of the other main challenges in understanding the role of biofilm development in persistence is the lack of standardised experimental protocols for comparing the biofilm formation of persistent and PNP strains. This has resulted in a wide range of different assays and experimental conditions being used and adds to the difficulty in comparing the results of different studies. In addition to making it difficult to determine whether persistent strains are more likely to exhibit enhanced biofilm formation, this also makes it difficult to identify the conditions under which they might do so. Therefore, there is a need for more standardised experimental protocols, particularly in relation to the choice of assay, temperature, surface contact time, growth medium and sample size, each of which may have an important bearing on a study’s ability to detect differences in biofilm phenotype [16,29,41]. For the reasons discussed, these should be tailored as far as possible to simulate the conditions encountered in different types of food-associated environments where persistence is known to occur, such as meat, fish and dairy processing plants [18].

## 8. Conclusions and Future Research Directions

Overall, it remains unclear as to whether enhanced biofilm formation contributes towards listerial persistence. While numerous phenotypic studies have been carried out to investigate this, the results have been highly conflicting. WGS studies, while limited in number, have also failed to identify any biofilm markers clearly linked to persistence. This may have been due, in part, to authors having relied upon different persistence definitions and/or different experimental protocols. Alternatively, it may be that biofilm formation is just one of several mechanisms that LM uses to persist, the importance of which may depend upon the strain, environmental conditions, or a complex interplay between the two. Clearly, a greater understanding of the mechanisms that LM uses to persist in food-associated environments is needed. However, for this to be achieved, further research is needed.

One of the most pressing research priorities is the need for a more standardised persistence definition, which would enable greater comparison between studies. For the reasons discussed, this should be based on a high-discriminatory subtyping method and include isolation after sanitation but before re-introduction of potential fomites. It should also incorporate checks to help rule out reintroduction from the outside environment, such as sampling of raw materials and adherence to GMP. One of the other main research needs is the need for more standardised protocols for comparing the biofilm formation of persistent and PNP strains. These should be tailored as far as possible to different types of food-associated environments and try to replicate the conditions there as accurately as possible, including the presence of food residues and other microorganisms. To this end, further studies should be undertaken to characterise the environmental conditions associated with listerial persistence. A greater understanding of these conditions and their effects on listerial biofilm development would help to shed light on the mechanisms that this organism uses to persist within food-associated environments. It may also help to identify ways of modifying the conditions within these environments in order to prevent persistence from occurring. Future biofilm studies should not just rely on crystal-violet staining but incorporate microscopy techniques capable of revealing biofilm morphology and techniques capable of quantifying the number of viable cells, such as plate counts.

The high throughput capability of WGS means it will continue to play an important role in studying bacterial genomics as it becomes quicker, cheaper and simpler to perform, due to advances in technology and bioinformatics [156]. As our understanding of listerial biofilm development mechanisms continues to develop, additional WGS comparisons of persistent strains should be conducted to try to explore whether particular listerial genotypes or genetic markers may be associated with persistence and, if so, under what conditions. Other ‘omic’ techniques, including transcriptomics, proteomics and metabolomics, are also offering exciting new insights into the behaviour of bacterial cells at the RNA, protein and metabolic levels, respectively [164]. For example, using transcriptomics, Fox et al. [165] identified several genes that were up-regulated in persistent compared with PNP LM strains isolated from cheese processing plants when cultured in the QAC disinfectant benzethonium chloride. These included the gene operons *pdu*, *cob*-*cbi* and *eut,* involved in ethanolamine and 1,2-propanediol metabolism. Similarly, using an exoproteome analysis, Rychli et al. [166] were able to identify 16 proteins potentially associated with persistent strains of LM, although none were known to play a role in biofilm development.

Further ‘omic’ analyses of persistent strains may help to identify patterns of gene expression or metabolic activity, which may help to explain how this organism is able to persist. These should be conducted under conditions representative of food-associated environments, including mixed-culture conditions, to help understand the effect of interactions with other species. Unravelling the enigma of what drives listerial persistence in food-associated environments will be key to reducing persistent product contamination and, ultimately, improving food safety. 

## Figures and Tables

**Table 1:** Genetic Determinants Involved in Biofilm Formation in LM.

Gene(s)	Product	Role in Biofilm Formation	Reference
*actA*	Actin-assembly-inducing protein precursor (ActA)	Promotes bacterial aggregation. Required to reach wild-type biofilm levels on glass under static and continuous-flow conditions.	[122]
*agrBDCA*	Peptide-based sensing system	Involved in early adherence to glass and polystyrene. May act via quorum sensing.	[131,132]
*bapL*	BapL, a putative cell wall-anchored protein	Involved in adherence to polystyrene and stainless steel (although potentially only in specific strains and serotypes). Mechanism is unclear.	[113,125,126]
*comK* prophage	N/a	*comK* prophage associated with enhanced biofilm formation on abiotic surfaces.	[139]
*degU*	DegU, a putative response regulator	Involved in adherence to polystyrene.	[120,121]
*flaA*	Flagellin A, the main structural component of bacterial flagella	Involved in initial adherence under static and dynamic conditions but may inhibit biofilm formation under dynamic conditions.	[114,115,116,117]
*inlA*	InlA, a cell surface protein important for virulence	Involved in adherence to glass. Truncation is associated with increased biofilm formation on polystyrene.	[127,128,129]
*inlB*	InlB, a cell surface protein important for virulence	Involved in adherence to glass.	[127,128]
*inlL*	InlL, a cell surface protein	Involved in initial adherence to polystyrene.	[130]
*lmo2504*	Lmo2504, a putative cell wall-binding protein	Unclear. Overexpressed in biofilm-associated, compared with planktonic cells.	[138]
*luxS*	LuxS, an AI-2 biosynthesis protein	Intact gene associated with repression of biofilm development on glass.	[135,136]
*prfA*	PrfA, a transcriptional regulator	Involved in the late stages of biofilm development.	[118,119]
*recO*	RecO, a DNA repair and protection protein	Overexpressed in biofilm-associated compared with planktonic cells.	[137]
*sigB*	SigB, a major transcriptional regulator of stress response genes	Required to obtain wild-type biofilm levels under static and continuous-flow conditions on stainless steel and polystyrene.	[123,124]

## Data Availability

No new data were created or analysed in this study. Data sharing is not applicable to this article.

## Supplementary Materials

The following supporting information can be downloaded at https://www.mdpi.com/article/10.3390/foods12183339/s1, Table S1: Summary of Studies Comparing the Biofilm Production of Persistent *Listeria monocytogenes* (LM) Strains Isolated from Food-Associated Environments to that of Presumed Non-Persistent (PNP) Strains. References [167,168,169,170,171,172,173] are cited in the supplementary materials.
