# Supplementary material for: Listeria monocytogenes Biofilms in Food-Associated Environments: A Persistent Enigma
Source: Foods. 2023 Sep 6;12(18):3339. doi: 10.3390/foods12183339 (PMC10529182; doi:10.3390/foods12183339)
Supplement: Supplementary file 1 [file foods-12-03339-s001.zip › foods-2513305-supplementary.pdf]

Supplementary Table S1: Summary of Studies Comparing the Biofilm Formation of Persistent and Presumed Non-Persistent (PNP) *Listeria monocytogenes* (LM) Strains Isolated from Food-Associated Environments.

Source of Persistent Isolates	Subtyping Method(s)	Persistence Definition	No of Persistent (and PNP) LM Isolates	Surface Material	Surface Contact Time (and Temp)	Growth Medium	Biofilm Assay	Key Findings	References
Miscellaneous food processing establishments and farm environments (Raw milk and non-dairy foodstuffs)	MEE, RFLP, serotyping	Strains recurring in sequential samples over a period of months from the same farm and food processing environments.	35(25)	Stainless steel	24 hrs (25 °C)	TSB (1:15)	Plate counts	Mean adherence of persistent strains significantly higher than that of PNP	[27,167]
Poultry and ice cream plants (Processing environment, equipment and	PFGE, serotyping	Dominating strain isolated repeatedly within the plant over several months or years.	3(14)	Stainless steel	1 and 2 hrs or<br>72 hrs<br>(25 °C)	TSB	Epifluorescence microscopy cell counts	Persistent strains exhibited significantly higher adherence compared with PNP strains after 1-2 hrs; after 72 hrs, 50 % of PNP strains had equal or greater adherence levels.	[28]

Fish processing plants<br><br>(Fish processing environment)	Ribotyping,<br><br>RFLP	Repeated isolation from the same processing plant.	5(5)	PVC	40 hrs<br><br>(32 °C)	MWB	Microtiter plate assay with crystal-violet staining	No relationship was observed between persistence and biofilm formation.	[56]
Meat dicing plants<br><br>(Dicing machine, dicing lines, diced products)	PFGE, serotyping	Not clearly defined (although persistent pulsotype isolated from one plant over a period of up to 16 months).	1(3)	Stainless steel	2 hrs (25 °C)	TSB	Epifluorescence microscopy cell counts	Persistent strain exhibited significantly higher adherence than the PNP strains.	[87]

Dairy processing plant<br><br>(Bulk milk samples)	PFGE	Repeated isolation of a given strain from bulk milk samples obtained from the same dairy.	11(15)<br><br>1(1)	PVC<br><br>Stainless steel and PVC	40 hrs (30°C)<br><br>40 hrs (30°C)	MWB<br><br>MWB	Microtiter plate assay with crystal-violet staining<br><br>Scanning electron microscopy	Persistent strains exhibited significantly higher mean biofilm formation compared with PNP strains.<br><br>The PNP strain produced sparse cellular aggregates on stainless steel and attached to PVC mostly as single cells. Conversely, the persistent strain produced dense, 3D cellular composite with well-distributed channels on both materials.	[29]
Miscellaneous food processing establishments and farm environments (Raw milk, cooked meat/poultry and processed vegetables)	MEE, ESE	Recurrent isolation for periods ranging from a few months to up to a year in raw milk and non-dairy foods obtained from the same manufacturer.	36(32)<br><br>NR	Polystyrene<br><br>Polystyrene	48 hrs (20°C)<br><br>14 days (20°C)	TSB<br><br>TSB	Microtiter plate assay with crystal-violet staining<br><br>Petri dish assay with crystal-violet staining	Persistent and PNP strains exhibited similar levels of mean biofilm formation after 48 hrs of surface contact in microtiter plates or 14 days in petri dishes.	[92,167]

Fish processing plants<br><br>(Cold smoked salmon, ham, smokehouse equipment, slaughterhouse equipment)	RAPD	Not clearly defined (although persistent strains described as 'frequently occurring').	10(8)<br><br>NR	Polystyrene<br><br>Polystyrene	48 hrs (37°C)<br><br>NR (15°C	TSB or LB broth (with 1 % glucose and up to 5 % NaCl)<br><br>TSB or LB broth (with 1 % glucose and up to 5 % NaCl)	Microtiter plate assay with crystal-violet staining<br><br>Microtiter plate assay with crystal-violet staining	No systematic differences in adherence between the persistent and other strains in either medium.	[93]
Mussel processing plants<br><br>(Raw product, bale store, packing, heating, opening, external area)	PFGE, serotyping	Detection more than once in the same factory over six months.	76(25)	PVC	48 hrs (30 °C	TSB-YE (with 0.6 % (w/v) yeast extract)	Microtiter plate assay with crystal-violet staining	Persistence of strains did not directly correlate with their biofilm forming ability.	[94]

Dairy farm<br><br>(Bulk tank milk, in-line milk filter, milk meter, flies, faeces, environmental, milking equipment)	PFGE	Isolation of the same PFGE-type on at least three sampling occasions. collected at intervals ranging from one week to six months.	8(9). Included four pred/perst strains, three persistent and nine PNP strains	PVC	48 hrs (32 °C)	TSB	Microtiter plate assay with crystal-violet staining	Pred/perst strains exhibited significantly higher adherence compared with persistent and PNP strains.	[40]
Food processing plants (details NR****)	NR	Isolation of the same strain on more than one sampling occasion from the same factory over a period of 12 months to four years.	11(10)<br><br>1(1)	Polystyrene<br><br>Stainless steel	120 and 144 hrs (10°C)<br>24 and 48 hrs (<br>20°C)<br>24 and 48 hrs (25°C)<br><br>48 hrs (25°C)	BHI<br><br>BHI (pH 4.7, 7.3 and 8.5)	Microtiter plate assay with crystal-violet staining<br><br>Microtiter plate assay with crystal-violet staining	None of the persistent strains exhibited significantly higher biofilm formation compared with the PNP strains.	[47]

Fish processing plant<br><br>(Processed and unprocessed fish, raw fish processing and slicing areas)	AFLP, RAPD	Not clearly defined (although persistent strains described as ‘dominant’ and isolated from the same plant over months or years).	2(5)	Glass, PVC and glass coated with beef extract, casein, homogenised or unhomogenised milk.	30 minutes (37 °C)	TSB	Inverted microscopy	The initial adherence rate of the strains did not generally relate to persistence when evaluated under liquid flow at two levels of shear stress on each surface material.	[26,95,168]
Cold-smoked fish processing plant<br><br>(Slicing machines)	Serotyping, PFGE and PCR	Most dominant clone isolated throughout the study period (2002-2004).	1(1)<br><br>1(1)<br><br>1(1)	PVC<br><br>PVC<br><br>Bakelite	16, 24, 40, 48 and 72 hrs (32 °C)<br><br>16, 24, 40, 48 and 72 hrs (32 °C)<br><br>72 hrs (32 °C)	MWB<br><br>MWB<br><br>MWB	Microtiter plate assay with crystal-violet staining<br><br>Plate counts<br><br>Phenol-sulphuric acid method	Persistent strain exhibited significantly greater biofilm formation than the PNP strain after 40 hrs or more surface contact, based on crystal-violet staining (but not plate counts), and significantly higher EM production on Bakelite after 48 hrs or more.	[111]

Retail deli sites<br><br>(Chicken meat samples)	MvLST and Serotyping	Repeated isolation of the same strains from chicken samples in the same retail site over six months.	30(25)	Polystyrene	48 hrs (30 °C or 37 °C)	MWB	Microtiter plate assay with crystal-violet staining	Persistent strains exhibited significantly higher mean biofilm formation than PNP strains at 37 °C but not 30 °C.	[102]
Iberian pork processing plant	PFGE	Isolation three or more times from the environment or equipment over at least three months.	8(21)	Polystyrene	48 hrs (37 °C)	TSB-YE (1 % glucose and 2 % NaCl)	Microtiter plate with crystal-violet staining	Surface attachment varied widely according to strain, with no clear relationship between persistence.	[157]
Retail delis<br><br>(Food contact surfaces, non-food contact surfaces and transfer points, such as deli case door handles and slicer knobs)	PFGE	Isolation three times from the same retail deli over a six-month period.	23(73)	PVC	1-5 days (30 °C)	HTM (with 3.0 % glucose)	Microtiter plate assay with crystal-violet staining	Persistent strains exhibited significantly higher adherence on day one of a five-day adherence-biofilm formation assay. However, there were no significant differences in biofilm formation on days three and five.	[152,169]

Gorgonzola cheese processing plants	PFGE	Isolation of strains with identical or highly similar (>95 %) pulsotypes during different years of the collection period (2004-2007).	11(5)	Polystyrene<br><br>Stainless steel	24 hrs (25°C)<br><br>48 hrs (25°C)	TSB-YE (with 0.6 % yeast extract)<br><br>TSB-YE (with 0.6 % yeast extract)	Microtiter plate assay with crystal-violet staining<br><br>Plate counts	No significant differences in biofilm formation were observed between persistent and PNP isolates, except on stainless steel (where higher cell counts were obtained for the PNP isolates).	[98,170]
Miscellaneous foods and food-processing environments in Europe<br><br>(Cheese, smoked salmon, blue-veined cheese, food processing plant)	PFGE, serotyping	Isolation from defined sites or samples on multiple occasions over 12 months or on multiple occasions from 2004-2010.	20(13)	Polystyrene<br><br>Polystyrene<br><br>Polystyrene	24 hrs (20, 37 or 40°C)<br><br>24 hrs (20, 37 or 40°C)<br><br>5 days (4°C)	TSB<br><br>TSB (1:10)<br><br>TSB (1:10)	Microtiter plate assay with crystal-violet staining<br><br>Microtiter plate assay with crystal-violet staining<br><br>Microtiter plate assay with crystal-violet staining	No significant differences in biofilm formation were detected between persistent and PNP strains under any of the test conditions	[97]

Cheese production plant<br><br>(Isolation site NR)	MLST, serotyping	Isolation on >50 % of sampling sites dates over two years ('frequent persisters').	1(10)	Polystyrene<br><br>Stainless steel	40 hrs (20 or 37°C) or 72 hrs (20°C)<br><br>72 hrs (20°C)	TSB (neat or diluted 1:10)<br><br>TSB (neat or diluted 1:10	Microtiter plate assay with crystal-violet staining and plate counts<br><br>Plate count	'Frequent persister' strain (6179) was one of the strongest biofilm-producers throughout the study.	[88,165]
Cheese processing plants<br><br>(Drain in cooling chamber, floor of cooling chamber, platform of cooling chamber, plastic crates, cheese)	PFGE	Isolation on at least two different sampling occasions from Nov 2008 to Sept 2009.	42(43)	Polystyrene	48 hrs (35 °C)	TSB	Microtiter plate assay with crystal-violet staining	Persistent isolates belonging to four pulsotypes were classed as weak/moderate biofilm-producers, while PNP isolates belonging to four separate pulsotypes were classed as strong biofilm-producers.	[98]

Cheese processing plants<br><br>(Cheese, shipping zone, table)	PFGE	Isolation of LM isolates with indistinguishable molecular PFGE types on different dates and origins over four years.	6(7)	Polystyrene<br><br>Stainless steel, silicon rubber and PVC	5 days (22°C)<br><br>5 days (22°C)	TSB<br><br>TSB	Microtiter plate assay with crystal-violet staining<br><br>Plate counts	Persistent strains exhibited significantly stronger biofilm formation on stainless steel and silicon rubber compared with PNP strains. No significant differences were observed on PVC or polystyrene.	[104,171,172]
Mussel processing plants<br><br>(Heating, external area (e.g. entrance doors/floors), opening, bale store)	PFGE	Six of the persistent strains detected more than once in the same factory over six months. Persistence of the other two strains not defined.	8(8)	Polystyrene<br><br>Polystyrene	24 and 48 hrs (20 °C)<br><br>24 and 48 hrs (30 °C)<br><br>24 and 48 hrs (20 °C)<br><br>24 and 48 hrs (30 °C)	BHI<br><br>BHI	Microtiter plate assay with crystal-violet staining.<br><br>Plate counts	Persistent strains exhibited stronger biofilm formation than PNP strains but the difference only reached statistical significance (based on plate counts) after 48 hrs at 30 °C.	[89,94]

Pig slaughterhouse<br><br>(Viscera tank, chilling door, saw, bleeding area and meat conveyor)	PFGE, WGS (cgMLST and hqSNP analysis)	Isolation from the same site after cleaning and disinfection on three sampling occasions over a month, 1-2 weeks apart.	25(5)	Polystyrene	48 hrs (30 °C)	BHI broth (with or without 0.78 ppm BC)	Microtiter plate assay with crystal-violet staining	No significant difference in biofilm formation between persistent and PNP strains was observed under any of the test conditions	[99]
Food processing plants<br><br>(Smoked salmon, catering plate, ground, food contact surfaces, food conveyor, chain bracket, sewer, slicer or other machine before cleaning, ham and butter sandwich, factory environment)	MLST	Isolation from the same food premise of the same genotype at least three times over an extended observation period 1-5 years.	19(39)<br><br>4(2)	Polystyrene<br><br>Polystyrene	24 hrs (10°C, 37°C)<br><br>5 hrs (10°C, 37°C)	BHI (neat or diluted 1:10; 0 or 0.85 % NaCl (w/v)<br><br>BHI (neat or diluted 1:10)	Microtiter plate assay with crystal-violet staining<br><br>Biofilm Ring Test	Persistent strains generally did not exhibit significantly higher biofilm formation under most test conditions compared with PNP (rare and prevalent) strains, except after 24 hrs in the microtiter plate assay containing neat BHI at 37°C (compared with prevalent isolates).	[44]

Seafood and poultry processing plants<br><br>(Non-food contact surfaces, including drains, floors and walls; food contact surfaces, including boxes, equipment and tables; and hamburger meat)	MvLST	NR	NR<br><br>NR	PVC<br><br>Stainless steel	24 hrs (37°C)<br><br>24 hrs (37°C)	NR<br><br>NR	Microtiter plate assay with crystal-violet staining<br><br>Plate counts	The most persistent strains (VT10, VT11 and VT94) formed biofilms on PVC. However, two of the PNP strains formed biofilms to a similar (VT8) or greater extent (VT6) as the persistent strains. None of the strains formed biofilms on stainless steel.	[160]
Chicken processing plant<br><br>(Product and non-product contact surfaces)	Ribotyping	Isolation of identical ribotypes on at least two sampling occasions, six months apart.	5(5)	Polystyrene	24 hrs (37 °C)	TSB	Microtiter plate assay with crystal-violet staining	Persistent strains showed significantly stronger biofilm formation than PNP strains.	[90]

Pig slaughterhouse<br><br>(Conveyor belts)	PFGE, serotyping	Recurrent isolation of a strain with an identical/similar PFGE-profile from the conveyor belt (final packing) between Sept 2016 and Jan 2017.	NR	Polystyrene	48 hrs (35 °C)	TSB	Microtiter plate assay with crystal-violet staining	All strains classified as weakly adherent.	[100]
Cold-smoked salmon processing plant<br><br>(Foods, food processing environment, other)	WGS (hqSNP analysis), ribotyiing and MLST	Isolation from the facility over more than a one-year period.	7(5)	Polystyrene	120 hrs (10 °C or 21 °C)	BHI	Microtiter plate assay with crystal-violet staining	Persistent (cluster three) isolates exhibited significantly higher attachment at 21 °C compared with PNP isolates (clusters one and two) but not at 10 °C.	[91,156]

Sushi processing plants<br><br>(Salmon sushi samples)	PFGE	Isolation on at least two sampling occasions from samples at the same plant, 37 days apart.	4(2)	Stainless steel	24 hrs (37 °C)	TSB	Plate counts	No significant differences in biofilm formation were observed between persistent and PNP strains.	[101]
Meat processing plant<br><br>(Production environment, raw beef)	cgMLST, wgMLST	Re-isolation from the same plant four years apart.	2(2)	Stainless steel	72 hrs (37 °C)	BHI with or without: curing salts (5, 7.5 or 10 % (w/v)); NaCl (5, 7.5 and 10 % (w/v); or quaternary ammonium compounds (1:1,024)	Microtiter plate assay with crystal-violet staining	Persistent strains exhibited similar or lower levels of adherence compared with PNP strains under most conditions. One persistent strain (19) exhibited markedly higher adherence than all other strains in neat BHI.	[103,173]

Salmon processing plant<br><br>(Trim conveyor rollers, cutting element of head remover machine, filleting machine knives, skin remover machine, trommel of pin bone remover)	PFGE	Strains representing genetically identical isolates obtained from a particular part of the processing line at least three times over six months.	6(6)	Stainless steel	1-5 hrs (37°C)	BHI	Plate counts	Persistent strains initiated biofilm formation significantly faster than PNP strains, except after five hrs of surface contact.	[19]
				Stainless steel	72 hrs (20, 37°C) or 12 days (4°C)	BHI 0.5, 1.0 or 1.5 medium (pH 4, 7 or 9; 0, 5 or 10 % NaCl)	Plate counts	Persistent strains formed significantly stronger biofilms than PNP strains, except in 0.5 and 1.5 BHI or 5 % salinity (although the persistent strains adhered in higher numbers).	

* AFLP=amplified fragment length polymorphism; cgMLST= core-genome multilocus sequence-typing; ESE=esterase electrophoresis; hqSNP=high-quality single nucleotide polymorphism; MEE=multilocus enzyme electrophoresis; MLST=multilocus sequence-typing; MvLST=multivirulence locus sequence-typing; PFGE=pulsed-field gel electrophoresis; RAPD=randomly amplified polymorphic DNA; RFLP=restriction fragment length polymorphism; wgMLST=whole-genome-multilocus sequence-typing; WGS=whole-genome sequencing.

** Table column includes medium type and where reported, dilution (prior to adding inoculum), pH and concentration of any additives. BHI=Brain Heart Infusion; BHI 0.5=BHI broth containing 50 % of the amount recommended by the manufacturer; BHI 1.0=BHI broth containing the amount recommended by the manufacturer; BHI 1.5=BHI broth containing 150 % of the amount recommended by the manufacturer; BHI-YE=BHI with Yeast Extract; HTM=Hsiang-Ning Tsai; LB=Luria-Bertani; Modified Welshimer's Broth (MWB); SNP=single nucleotide polymorphism; TSB= Tryptic Soy Broth; TSB-YE=TSB with Yeast Extract

***Benzalkonium chloride

****NR=Not reported
